# Lessons learnt from a primary care asthma improvement project

**DOI:** 10.1038/npjpcrm.2015.75

**Published:** 2016-01-07

**Authors:** Warren Lenney, Sadie Clayton, Francis J Gilchrist, David Price, Iain Small, Judy Smith, Emma J Sutton

**Affiliations:** 1 Institute of Science and Technology in Medicine (ISTM), Keele University, Stoke-on-Trent, UK; 2 Department of Child Health, Royal Stoke University Hospital (RSUH), Stoke-on-Trent, UK; 3 Department of Primary Care Respiratory Medicine, Aberdeen University, Aberdeen, UK; 4 Respiratory MCN, NHS Grampian; 5 British Lung Foundation, London, UK; 6 North Staffordshire CCG and Stoke-on-Trent CCG, Staffordshire, UK

## Abstract

Asthma is a very common disease that can occur at any age. In the UK and in many other countries it is mainly managed in primary care. The published evidence suggests that the key to improving diagnosis and management lies in better training and education rather than in the discovery of new medications. An asthma improvement project managed through the British Lung Foundation is attempting to do this. The project has three pilot sites: two in England supported by the Department of Health and one in Scotland supported by the Scottish Government. If the project is successful it will be rolled out to other health areas within the UK. The results of this project are not yet available. This article highlights the challenges encountered in setting up the project and may well be applicable to other areas in the UK and to other countries where similar healthcare systems exist. The encountered challenges reflect the complex nature of healthcare systems and electronic data capture in primary care. We discuss the differences between general practices in their ability and willingness to support the project, the training and education of their staff on asthma management, governance issues in relation to information technology systems, and the quality of data capture. Virtually all the challenges have now been overcome, but discussing them should ensure that others become aware of them at an early stage should they wish to undertake similar projects in the future.

## Introduction

Data from many countries across the world continue to show that the diagnosis of asthma is of low quality^1–4^ and asthma remains poorly controlled.^5,6^ In countries with a high asthma prevalence,^7,8^ this poor control results in a significant number of asthma-related deaths.^9^ The recent UK National Review of Asthma Deaths has confirmed that many of these are potentially avoidable.^10^ The 10-year asthma programme in Finland showed that informing, teaching and educating healthcare workers, patients and families can lead to improved diagnostic accuracy of asthma, together with major reductions in both hospital admissions and asthma-related deaths.^11,12^ The Department of Health and the Scottish Government have funded a pilot project through the British Lung Foundation to determine whether an asthma improvement programme based on the Finland model can improve asthma control here in the UK. The project involves the training and education of doctors, nurses and other healthcare workers in three primary care sites (two in England and one in Scotland). The aim of the project is to improve the diagnosis and long-term management of asthma in children and adults. In the years since the Finland Project was completed, primary care has become increasingly dependent on electronic medical records.^13,14^ The project therefore necessitated the interrogation of primary care IT systems to analyse patient data and to provide bespoke reports with recommendations to each general practice about the management of individual patients cared for with a diagnosis of asthma.

## Aims of this article

The aim of this article is not to present the results of the Asthma Improvement Project as these are not yet available. The aim is to highlight the issues encountered in setting up this project in our three pilot sites and give a perspective on how these could be minimised if it is to be rolled out in other UK locations, or indeed in other countries.

## Methodology of the primary care asthma improvement project

Patient data were extracted immediately before the commencement of the educational aspects of the project. A social enterprise organisation well versed in remote extraction/capture and interpretation of international GP practice data was used to facilitate this process. To gain additional subjective patient information on asthma self-management and control, we planned to send a postal questionnaire to all asthma patients. Because of the difficulties with information governance as discussed below, this was only achieved in 14 practices within one pilot site. The data made available from the electronic extraction and the questionnaires were then used to generate individual reports for each practice. Each report summarised the management and control of all asthma patients at that practice as well as highlighting patients at high risk. These high-risk patients included those who were not on a preventer inhaler but who were collecting large numbers of prescriptions for reliever treatment. Other high-risk patients were those at BTS Step 4–5 of the National Guideline for Asthma Management^15^ but not being followed up in secondary care.

The initial data extraction and practice report generation has been successfully completed, as has the educational programme. We are in the process of undertaking a second data extraction and postal questionnaire distribution. This will take a number of months. The first and second sets of data will be compared to allow objective assessment of the usefulness of the project. If it is successful in improving asthma management it will be rolled out across the UK and we believe the results may well be applicable to similar projects in other countries.

## Heterogeneity between general practices

Although we work in a National Health Service our pilot projects have discovered major differences between sites in relation to the IT system used, how diseases are coded, the quality of the data collected, and the methods of governance employed from practice to practice.^16,17^ The three pilot sites had widely differing practices that have impacted on the implementation of the project on the data extraction. Some practice managers and clinicians have been more supportive of the project than others, which has determined how easily the project team has been able to interact together and arrange training sessions with other practice team members. Initially, some GP practices were wary of data being extracted by an external organisation, even though it was clarified that the data would be fully anonymised. There were additional concerns that the verification of individual patient names and their correct present addresses before the sending out of questionnaires would be time-consuming and that practice staff may not have sufficient time to do this.

## Local financial incentives affecting project participation

In the UK, the Primary Care Quality Outcomes Framework enables a level of payment to be received by the practice if patients with asthma receive an annual review.^18,19^ In England, Clinical Commissioning Groups (groups of GP practices involved in the commissioning of clinical services) can also include asthma within their local quality incentive schemes, which are locally agreed quality drivers with additional financial incentives that could, for example, include a more detailed asthma review.^20^ We recognised that we could adapt this to become part of our pilot project. The system in Scotland is somewhat different. Only one of the three pilot sites had fully included asthma into its incentivised quality improvement scheme resulting in all of its 33 practices participating in the project. The uptake in the other two sites was significantly lower, with only 18 of 52 (34.6%) and 21 of 78 (26.9%) practices participating. The poor uptake in these two areas delayed timely commencement and ultimately reduced the numbers of patients participating.

## Different information technology systems

The National Health Service has failed to develop a universal IT system with ‘National Health Service Connecting for Health’ being disbanded in 2013.^21^ This has resulted in the development of many different IT systems within the primary care setting alone.^22^ In our pilot project we discovered at least 5 completely different systems. Each system used different codes for the same disease states and different codes for patients who had requested that their data not be interrogated. Individual programme templates therefore had to be written for each IT system to extract the relevant data and ensure that patients not wanting their data interrogated were excluded from the project. One IT system did not allow any form of remote data extraction and hence it became necessary for a senior project team member to visit each of those individual general practices using that IT system to extract the data at source, taking up to three days per practice. Even when data could be extracted remotely, the smooth running of this placed a significant workload on the local IT teams, which was not envisaged before the project commencement.

## Information governance

After obtaining approval from the relevant Caldicott Guardians,^22^ the first data extraction generated a list of patients with asthma to whom the postal questionnaires would be sent. Before this, patients who had requested that their data not be interrogated needed to be excluded from the project. We discovered that up to 24 different exclusion codes were being used for this purpose alone depending on which IT system was used. Because of a time lag between checking exclusion codes and the first batch of questionnaires being sent (from 14 general practices), a small number of questionnaires were unfortunately sent to patients who had requested exclusion, resulting in one written complaint. The complaint was satisfactorily dealt with but a decision was made to abandon further posting of the initial questionnaires. We do hope, however, to send questionnaires to all patients at completion of the project if we are fully confident by then that all loopholes have been eliminated.

## Variation in training and education of practice staff

To enable planning of the educational programme, a questionnaire was completed by practice staff to identify their training needs. This highlighted significant variation between practices and individuals in relation to what training they had received and to their understanding of asthma and its management. It was also observed that it was not always a trained doctor or nurse who undertook parts of the patient’s asthma review, such as assessment of inhaler technique and the completion of a Personal Asthma Action Plan.^23^ The training programme therefore had to be tailored to the needs of individual practices and the needs of their specific healthcare professionals. Common areas highlighted for additional training included spirometric lung function testing and the management of children, especially those of preschool age. In general, the younger the child, the less confident staff were in the diagnosis and management of the child’s symptoms. This replicates findings of previous studies, which have also demonstrated that confidence can be increased with appropriate training and education.^24^ Other studies have highlighted how to improve understanding and interpretation of spirometric tests.^25–27^ Once key practice staff received their initial training they were expected to disseminate this to other practice staff as a pre-planned cascade process. This process has been variable depending on communication within practices and lack of time to take on additional work. There was also wide variation between practices in the availability of patient information sheets, placebo inhalers, peak flow metres and written asthma action plans. We therefore developed a comprehensive asthma toolkit containing all of the above-mentioned items. It was distributed to each practice with good feedback on its contents and helpfulness. We will evaluate the toolkit at the end of the project and if it proves successful it will be available through the BLF for others to use by the end of 2015.

## Data quality

The quality of data extracted is clearly dependent on the quality inputted.^28^ To meet Quality Outcomes Framework criteria, practices must undertake an annual review of each patient with asthma, but all that is essentially required is that a review has taken place. No specific details regarding what actually took place during the asthma review are required to be documented. As part of this project, a detailed asthma review checklist has been included in the IT systems. We expect that this will improve the quality of the annual review and the quality of the clinical information that is recorded.

After initial data extraction, individualised reports were produced for each practice as discussed above. Some of the initial summary data in these reports were inaccurate because of the various and different codes used in the extraction process. This led to lengthy discussions within a few practices resulting in a temporary loss of confidence in some primary care colleagues about the accuracy of any extracted data. For practices to view reports generated on individual patients, a desktop toolkit was needed. There have also been issues in using this toolkit, some practices being unable to access individual patient data. All these issues were unexpected but have now been rectified. To achieve this, however, has taken considerable time.

## Secondary care issues

Although this project is concentrated in primary care we have also discovered service issues within the hospital setting. Some adult patients with asthma referred for a secondary care opinion have waited an unacceptable length of time before being seen because of lack of out-patient slots. Those with more severe asthma (BTS/SIGN Steps 4–5) have not always been followed up in secondary care because of a lack of capacity within the hospital adult asthma service. Both children and adults admitted to hospital with an acute attack of asthma have, at times, been discharged without a Personal Asthma Action Plan.

## Conclusions

Despite national guidelines being available for the diagnosis and management of asthma since 1993,^29^ the quality of the diagnosis remains poor^1–4^ and patients continue to die unnecessarily.^10^ Key recommendations from the National Review of Asthma Deaths include better education for patients, parents and healthcare professionals, better monitoring of asthma control, and the provision of a Personal Asthma Action Plan for all patients. This pilot project aims to improve adherence to national and international guidelines and address the key recommendations of the National Review of Asthma Deaths. Although only performed in three primary care sites, our project has encountered many challenges in undertaking data extraction and the setting up of an asthma education and training programme. Many of the above challenges are broad and not specific to asthma. We hope that this will arm future researchers with information on, and understanding of, the challenges they may well encounter. We hope that this will help similar future studies in asthma or other disease states in the UK and in other coutries.[Boxed-text box1]

## Fundamentals for successful projects in primary care

1. Befriend the practice managers. They are crucial for project success.
2. Obtain ‘buy-in’ from all practice staff for remote interrogation of data.
3. Know and understand which IT system is used in each practice.
4. Select practices with the same IT system or expect complications.
5. Know the specific disease codes and exclusion codes for each IT system.
6. Recognise the need for financial compensation in primary care practices.
7. Practice staff have little time available for project work.
8. Local incentivised quality improvement schemes can be huge drivers for maximal practice participation.
9. Service and training standards vary widely between practices.
10. Bridge the divide between the primary community and the secondary hospital workforce by working together and understanding the issues involved.
